# Trehalose in cryopreservation. Applications, mechanisms and intracellular delivery opportunities

**DOI:** 10.1039/d4md00174e

**Published:** 2024-07-19

**Authors:** Alex Murray, Peter Kilbride, Matthew I. Gibson

**Affiliations:** a Department of Chemistry, University of Warwick CV4 7AL UK; b Division of Biomedical Sciences, Warwick Medical School, University of Warwick CV4 7AL UK; c Asymptote, Cytiva Chivers Way Cambridge CB24 9BZ USA; d Department of Chemistry, University of Manchester Oxford Road Manchester M13 9PL UK Matt.gibson@manchester.ac.uk; e Manchester Institute of Biotechnology, University of Manchester 131 Princess Street Manchester M1 7DN UK

## Abstract

Cryopreservation is crucial to fields including immune and stem cell therapies, reproductive technology, blood banking, regenerative medicine and across all biotechnology. During cryopreservation, cryoprotectants are essential to protect cells from the damage caused by exposure to freezing temperatures. The most common penetrating cryoprotectants, such as DMSO and glycerol do not give full recovery and have a cytotoxicity limit on the concentration which can be applied. The non-reducing disaccharide trehalose has been widely explored and used to supplement these, inspired by its use in nature to aid survival at extreme temperatures and/or desiccation. However, trehalose has challenges to its use, particular its low membrane permeability, and how its protective role compares to other sugars. Here we review the application of trehalose and its reported benefit and seek to show where chemical tools can improve its function. In particular, we highlight emerging chemical methods to deliver (as cargo, or *via* selective permeation) into the intracellular space. This includes encapsulation, cell penetrating peptides or (selective) modification of hydroxyls on trehalose.

## Abstract 2

The state of the art, challenges and opportunities for chemists to engineer and deliver trehalose as a cryoprotectant to address challenges in biomedicine and biotechnology are reviewed.

## Graphical abstract

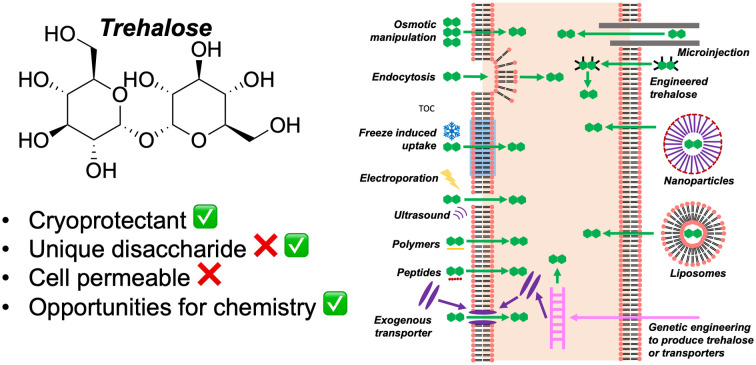

## Introduction

Cryopreservation is the practice of storing biological materials at sub-zero temperatures to halt metabolism and degradation. Cryopreservation is deployed for a vast range of biological materials from proteins,^1^ cells,^2^ tissues,^3,4^ mRNA vaccines,^5^ and potentially organs.^6,7^ Cryopreservation is essential in cell culture as it allows for the routine storage of cell lines, reducing the need for continuous culture, which would otherwise lead to phenotype drift as well as being practically challenging. It also has medical applications such as transporting stem cells,^8^ and blood for transfusion.^9^ Current cryopreservation techniques, whilst successful, are not perfect: a proportion of cells always die during the process and their functionality is often reduced. One of the major limiting factors in cryopreservation is cryoprotectant toxicity; cryopreservation requires the use of cryoprotectants (including but not limited to solvents) such as DMSO or glycerol which are penetrating cryoprotectants. These can have adverse effects when the thawed cells are transplanted into patients.^10^ Cryoprotectant toxicity also limits the concentration of cryoprotectant that can be used. The mechanism of cryoprotectant action is concentration-dependent but there becomes a point where further increases in concentration lead to toxicity that outweighs any protective effects. This has motivated research into non-/lower toxicity cryoprotectants.

**Fig. 1:**
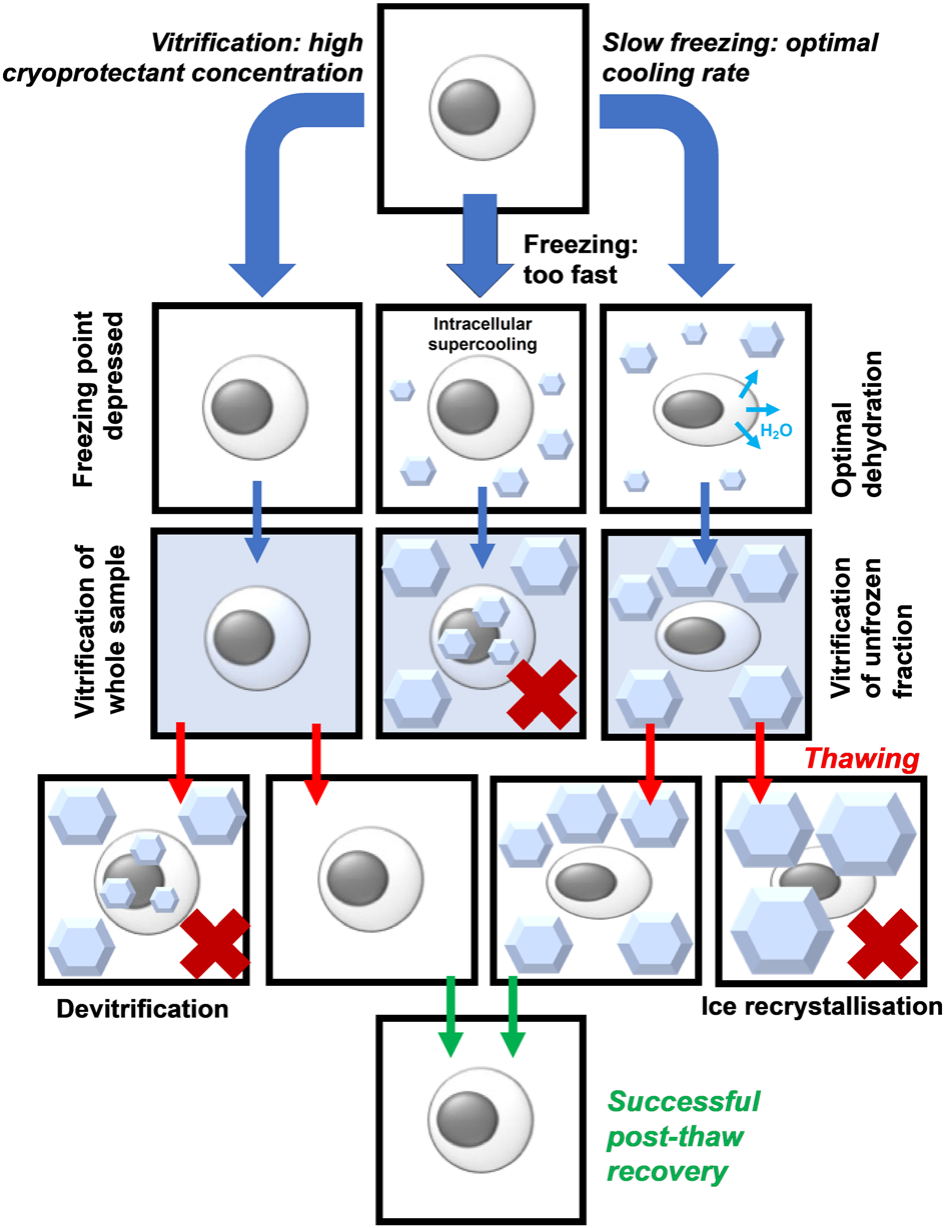
Schematic of the vitrification verses slow freezing pathways, indicating where failure can occur.

**Fig. 2:**
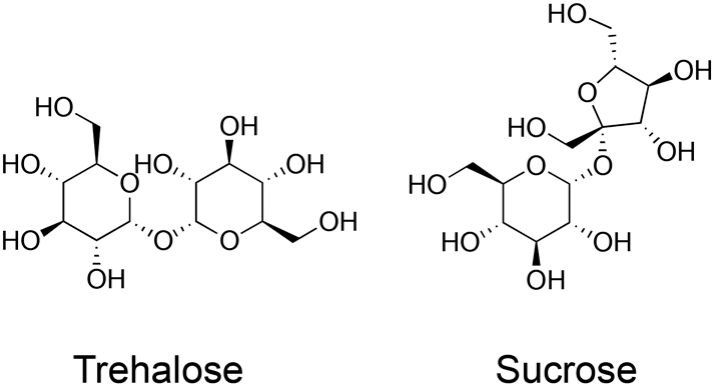
Chemical structure of trehalose and sucrose.

Trehalose (α-d-glucopyranosyl-(1 → 1)-α-d-glucopyranoside) is a water-soluble non-reducing disaccharide made up of two glucose subunits, joined by a 1,1-glycosidic bond.^11^ It has 8 hydrogen bond donors and 11 hydrogen bond acceptors. Trehalose is biosynthesised by a wide variety of non-mammalian organisms as an energy source and is used in some organisms to protect against freezing and desiccation, allowing them to survive over winter or in other harsh environments. These organisms include the rice water weevil *Lissorhoptrus oryzophilus*,^12^ the codling moth *Cydia pomonella*^13^ and some tardigrade species such as *Macrobiotus richtersi*.^14^ In the past, trehalose was expensive to make, but the development of an efficient manufacturing process in 1994 made this much cheaper, leading to increased research interest.^15^ Trehalose has two proposed protective mechanisms, which have been termed the ‘vitrification hypothesis’ and the ‘water replacement hypothesis’. These mechanisms are usually discussed in the context of preservation by desiccation or anhydrobiosis in nature but are also applicable to cryopreservation. It is crucial to note that trehalose (and other disaccharides) are non-penetrating cryoprotectants in contrast to the widely used DMSO or glycerol. Fig. 1 summarises the two key methods in cryopreservation relating to vitrification and slow-freezing, which necessitate different concentrations of cryoprotectant and freezing rates, and where problems can occur. Fig. 2 shows the chemical structure of trehalose compared to sucrose.

### The vitrification hypothesis

The vitrification hypothesis holds that trehalose protects biological materials by forming a high-viscosity glass-like state. The transition of a liquid trehalose solution to a glassy state is achieved by concentrating the trehalose through either desiccation or freeze-induced dehydration. During cryopreservation by freezing, the cells survive within the vitrified fraction of the sample.^16^ Trehalose has several properties that contribute to the formation of this fraction: adding trehalose to a solution increases its glass transition temperature (*T*_g_),^17^ resulting in vitrification at a higher temperature. Trehalose is also a kosmotrope,^18^ it orders water molecules around itself, altering the structure of the surrounding hydrogen bond network in such a way that it cannot form ice.^19^ This network is over three hydration shells wide,^20^ so each molecule of trehalose can prevent many molecules of water from freezing.^21^ Trehalose has been found to reduce the size of ice crystals in a concentration-dependent manner.^22^

### The water replacement hypothesis

**Fig. 3:**
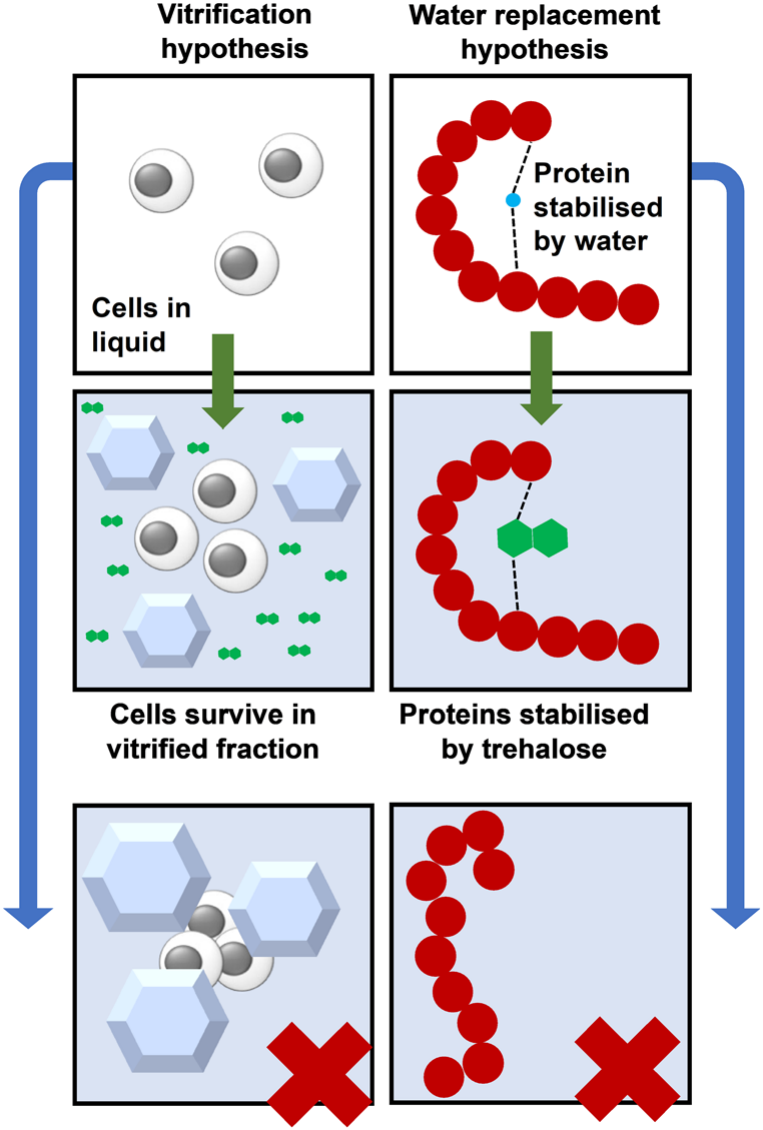
Vitrification compared to water replacement for protein storage using trehalose. The bottom panels show ice formation and protein denaturation in the absence of trehalose.

Under normal conditions, proteins and other cell components are stabilised/hydrated by bound water. The water replacement hypothesis holds that trehalose stabilises these components once the water content is sufficiently low by acting as a replacement for this water, Fig. 3.^23,24^ During cryopreservation, trehalose protects proteins from cold denaturation,^25^ and cryoprotectant toxicity.^26^ Trehalose also lowers the temperature at which the membrane gel-to-liquid crystal phase transition occurs.^27,28^ During desiccation, trehalose stabilises the cell membrane by hydrogen bonding to phospholipids,^29^ so trehalose may play a similar role in stabilising the cell membrane during cryopreservation. In addition to stabilisation, trehalose likely also counteracts solute concentration damage when it is used at sufficient concentrations, as would any biocompatible, water-soluble, small molecule.

Trehalose is a polar molecule (log *P* ∼ −3.7), which alongside its large number of hydrogen bond donors/acceptors, means it does not cross the cell plasma membrane and hence cannot provide intracellular cryoprotection. Thus, it is usually used as an extracellular cryoprotectant, but recent advances now allow its intracellular delivery. This review will firstly discuss trehalose as a cryoprotectant for different cell types and provide a critical comparison to other sugars, highlighting cases where trehalose does not offer additional benefit. Secondly, methods to deliver trehalose into the intracellular space and the impact on cryopreservation will be explored.

## Trehalose as a (primarily) extracellular cryoprotectant

The need for improved cryoprotectants has resulted in research into sugars as a supplement or replacement for traditional penetrating cryoprotectants such as DMSO, ethylene glycol, propylene glycol and glycerol. Trehalose has gained particular attention due to its unique properties among the sugars, which will be discussed in more detail later.

Trehalose has been tested as a cryoprotectant in an extensive range of cell types and other biological materials. In most studies, trehalose is supplemented into the standard penetrating cryoprotectant for that cell type – typically 10% DMSO. It is observed that this increases cell recovery and/or improves post-thaw proliferation and other functional outcomes. A recurring trend throughout these studies is that trehalose has an optimal concentration – usually between 100 mM to 400 mM – beyond which adding further trehalose becomes detrimental to post-thaw outcome.^30–32^ The detrimental effect of higher concentrations is likely due to osmotic pressure rather than through any specific trehalose toxicity. Importantly, trehalose can often lower the amount of penetrating cryoprotectant required for cryopreservation while providing an equivalent or better post-thaw outcome.

**Fig. 4:**
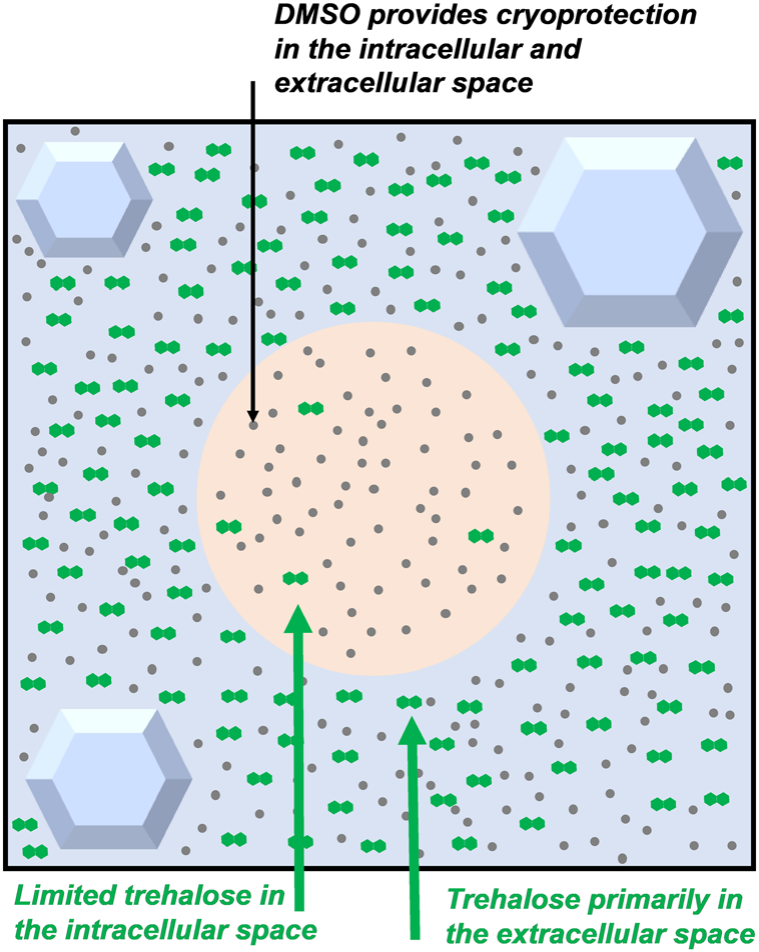
Schematic of localisation of trehalose compared to DMSO during cryopreservation (green hexagons = trehalose, grey dots = DMSO).

**Fig. 5:**
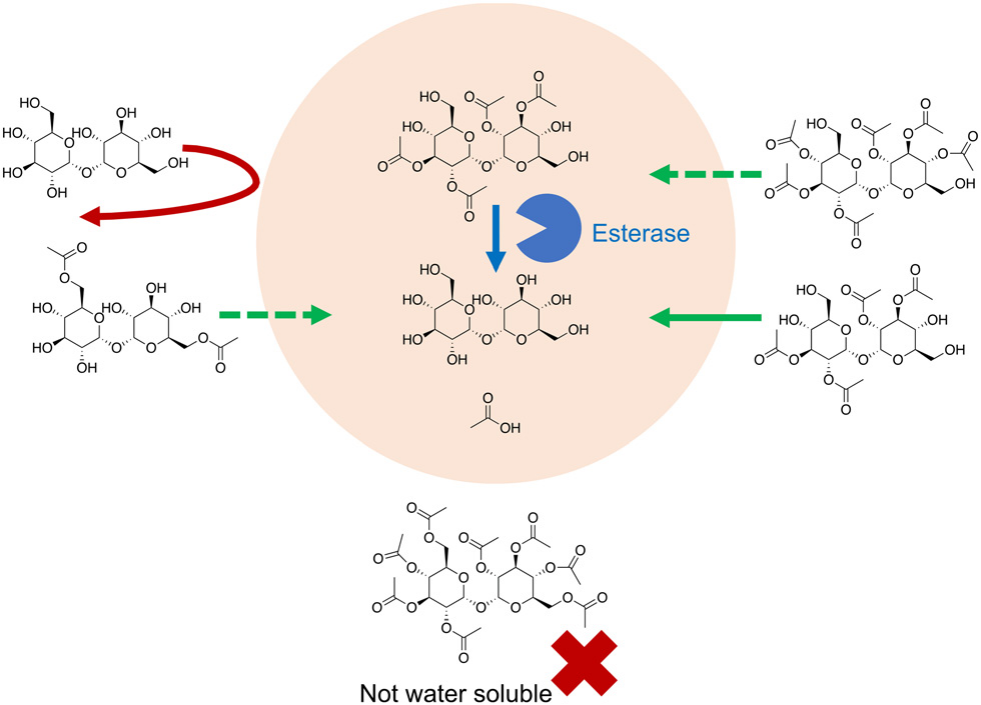
Possible mechanisms by which trehalose can enter cells.

**Fig. 6:**
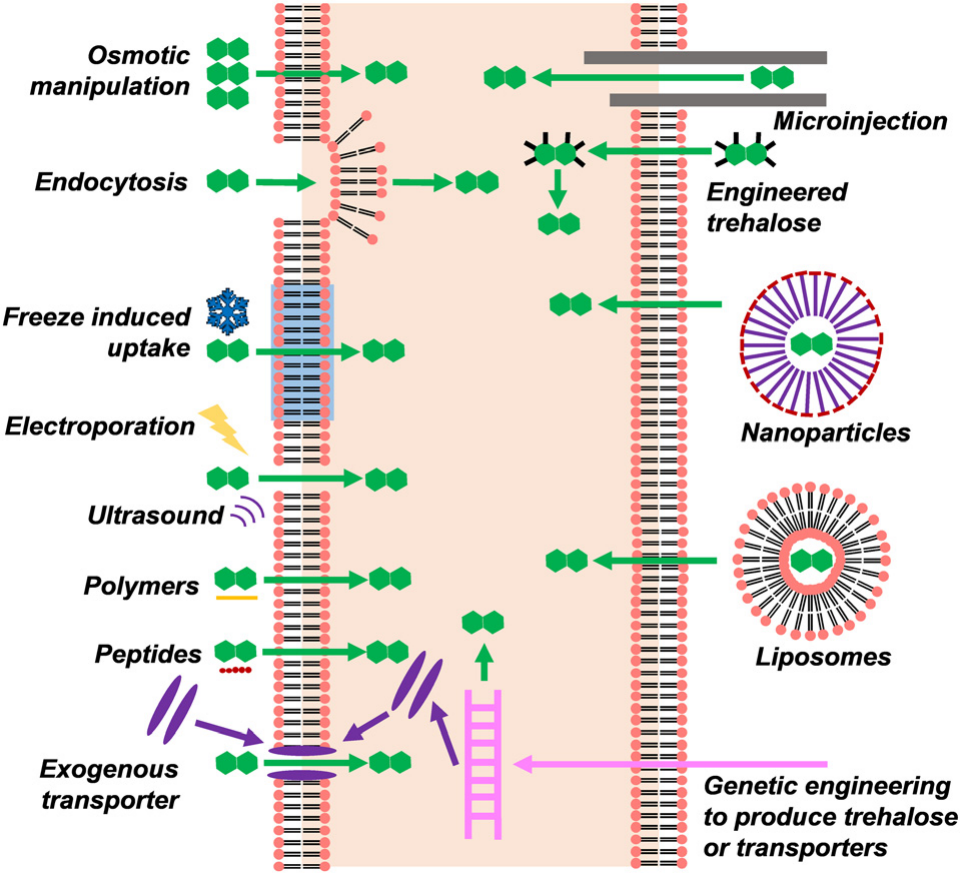
Engineered and formulated trehalose increases passive uptake by membrane diffusion followed by esterase action.

Trehalose is not cell penetrative and cannot normally cross the cell membranes and enter the intracellular space unaided, but occasionally, extracellular trehalose can be used without solvent cryoprotectants,^33–35^ suggesting the presence of intracellular trehalose, which can enter the cell through endocytosis or a membrane phase transition during cooling. Whilst this section is about the extracellular use of trehalose, small amounts of trehalose are likely to be present intracellularly in many of these studies. These concepts are discussed in later in this review. Fig. 4 shows an overview of this, and Fig. 5 and 6 demonstrate the individual mechanisms. A summary of trehalose supplementation to extracellular media and its impact is made below spanning a range of cryopreservation scenarios to allow the reader to compare and contrast the benefits.

### Stem cell preservation

For murine (mouse) spermatogonial stem cells, adding 50 mM trehalose to the standard 10% DMSO freezing media improved viability after storage for one week (90% *vs.* 76%).^36^ Adding 200 mM trehalose did not improve viability, but did improve long-term proliferation (49% *vs.* 28%). For murine microencapsulated mesenchymal stem cells, 5% DMSO, 10%, glycerol, 10%, 5% trehalose, or any combination of trehalose and less than 10% DMSO, all resulted in lower recovery than 10% DMSO.^37^ For mesenchymal stem cells DMSO can be partially replaced with trehalose or poly(ethylene glycol) (PEG), although trehalose was not more effective than PEG, which might suggest that lower DMSO was tolerated in this model.^38^

Human pluripotent stem cells: cryopreservation can be improved by replacing the standard 10% DMSO with 500 mM trehalose and 10% glycerol. Relative viability was increased by approximately 20–30% depending on the exact cell type. Phenotype and functionality were maintained.^39^ This raises a particular point that DMSO-free cryopreservation is possible, but in many cases achieved by adding a different penetrating solvent in its place.

Human umbilical cord blood stem cells: in Chen *et al.*^30^ 2.5% DMSO and 30 mM trehalose was at least as good as 10% EG and 2% DMSO, or 10% DMSO and 2.0% dextran-40 at maintaining viability. Trehalose also slightly reduced the amount of post-thaw apoptosis. In Zhang *et al.*^40^ freezing mediums containing 146 mM trehalose + 10% or 5% DMSO resulted in proliferation that was as high as the non-frozen control group. Trehalose has also been successfully used in combination with taurine and catalase for cord blood stem cell preservation.^41^

Human peripheral blood stem cells: in Martinetti *et al.*^35^ the use of 1 M trehalose alone gave higher long- and short-term viability than DMSO alone, or DMSO combined with trehalose. The 1 M trehalose concentration used here is much higher than the typical optimal concentration of 100–400 mM in most cell types; it is possible that the slow addition of freezing media to the cells in this study allowed a higher concentration of trehalose to be reached without causing too much osmotic stress. For marrow and blood stem cells Scheinkönig *et al.*^42^ found that 500 mM trehalose provides a similar level of cryoprotection to DMSO.

Human adipose-derived stem cells: cryopreservation with 250 mM trehalose alone resulted in very poor viability compared to 5% DMSO (11% *vs.* 75%).^43^ In Pu *et al.*^32^ 200 mM trehalose allowed the reduction of DMSO to 3.3% while maintaining good cryoprotection.

### Spermatozoa

Spermatozoa are distinct from other cell types in that they have a much larger surface area to volume ratio, have a more densely packed cytoplasm, and contain less freezable water, which makes them less vulnerable to intracellular ice formation.^44^ Thus, optimal spermatozoa cryopreservation differs from that of more typical cells, with protocols often preferring fast freezing or vitrification to slow freezing.^45^ Trehalose has been added to the “freezing extender” (a term referring to a freezing media used for spermatozoa) and tested in the cryopreservation of spermatozoa from many different animals.

Rabbit: spermatozoa vitrification with 4% DMSO was improved with the addition of 100 mM trehalose as measured by increased motility (43% *vs.* 25%), membrane integrity (45% *vs.* 24%) and mitochondrial membrane integrity (53% *vs.* 28%). Trehalose also decreased ROS and improved catalase function.^46^

Ram: post-thaw outcome was improved in multiple studies, with the optimal trehalose concertation typically being 50–100 mM.^47–53^ Some studies found that higher concentrations of around 200 mM are ineffective,^54,55^ while others have found positive effects at higher concentrations.^56,57^ These differences may be due to the total osmolality of the solution; in an extender with a high initial osmolality, less trehalose can be added before the total osmolality becomes too high and dehydrates the cells. Trehalose has also been combined with low-density lipoprotein for use as an extender.^58^

Equine: trehalose has had mixed results in equine spermatozoa, one study reported a benefit to using a small amount of trehalose.^59^ Other studies reported that trehalose does not improve post-thaw outcome.^60–62^

Other animals: trehalose also improved cryopreservation outcomes for the spermatozoa of goats,^63,64^ carp,^65^ and oysters.^66^ Trehalose did not provide benefit for the cryopreservation of European brown hare spermatozoa.^67^

### Tissue cryopreservation

Tissue cryopreservation is significantly more challenging than for individual cells (or *e.g.* spheroids) for several reasons. The large volume to surface area to ratio makes it difficult for a cryoprotectant to homogeneously permeate the tissue, and to ensure a uniform cooling rate. Extracellular ice formation is particularly damaging to the tissue macrostructure – unlike individual cells which can move freely within the media, allowing them to fit within the unfrozen portion of the sample. Several studies have found a cryoprotective benefit to using trehalose: in one study, the viability of adipose tissue was increased by 80% through the addition of 250 mM trehalose to an unspecified standard freezing media.^68^ In another study, the addition of 500 mM trehalose to 10% DMSO increased the post-thaw live cell count of human foetal skin (65% *vs.* 44%), improved post-thaw morphology, graft necrosis, and shrinking.^69^ Trehalose can also reduce post-thaw tissue pigmentation.^70^ Dog tracheas have been preserved for transplant in 10% DMSO and 100 mM trehalose, resulting in a 100% animal and graft survival rate (*n* = 6) after 184 days when transplanted. This study did not report cell recovery or viability.^71^ The viability of bovine calf testicular tissue, cryopreserved with 10% DMSO, was higher with increasing trehalose concentrations of up to 438 mM.^72^

### Other cell types

Human: viability of hepatocytes was improved (63% *vs.* 47%) with the addition of 200 mM trehalose to 10% DMSO.^73^ Trehalose and salidroside increased the survival of red blood cells (RBCs) after cryopreservation. However, adding salidroside to glycerol is slightly more effective than replacing glycerol with trehalose 61% *vs.* 56% respectively.^73^ Trehalose and catalase protect the proteins on the surface of human hematopoietic cells.^74^

Non-human: in murine embryos, 1.5 M glycerol and 100 mM trehalose was the optimal cryoprotectant combination for freezing. Interestingly at 2.0 M glycerol, increasing trehalose concentration increases viability in a dose-dependent manner, even beyond the optimal trehalose concentration at optimal and suboptimal glycerol concentrations. This suggests that glycerol prevents damage by preventing dehydration damage, and/or trehalose protects cells from glycerol toxicity.^75^ For *Trypanosoma brucei*, studies have found that 400 mM trehalose in host blood, or 200 mM trehalose *in vitro*, without DMSO or glycerol, is optimal for cryopreservation. In these studies, the replacement of 7.5% DMSO with trehalose alone resulted in drastic increases in survival rate, depending on the form of the trypanosome.^76,77^ In porcine spermatogonia stem cells, 200 mM trehalose alone, compared to 10% DMSO, did not significantly increase the recovery of testis cells, but did increase proliferation capacity and the recovery of germ cells from thawed testis cells and tissue.^78^ In ecto-mycorrhizal basidiomycetes, trehalose addition to DMSO improves post-thaw viability of most sub-types.^79^ Finally, trehalose has been used to cryopreserve multiple cell types by pre-freeze dehydration.^80^

#### Comparison with other sugars

The key question raised from the above is to what extent is trehalose unique among the sugars? Has trehalose proven beneficial as a cryoprotectant only to the extent that any sugar can act as a cryoprotectant? A particular advantage that trehalose has, compared to other disaccharides is that it is non-reducing and does not have a hemiacetal/aldehyde equilibrium, hence it cannot undergo associated side reactions with *e.g.* protein side chains. Furthermore, the glycosidic bond of trehalose is more stable than that of sucrose and is less susceptible to hydrolysis.^81^ Disaccharides in general also appear to be superior cryoprotectants than monosaccharides.^82,83^ Although, this is not always the case: in some studies, the monosaccharides galactose^84^ and inositol^85^ have been more effective than disaccharides. One explanation for the relative success of disaccharides could be that, at any given molarity, disaccharides have more polar groups while exerting the same osmotic pressure. Thus one important consideration when comparing monosaccharides with disaccharides is that the experimental concentrations of sugars in cryoprotectant solutions are usually measured in terms of molarity, rather than % weight; comparing 100 mM monosaccharide with 100 mM disaccharide can be misleading because the former tend to be approximately half the molecular weight of the latter, and critically – contain just over half as many polar groups per mol, resulting in fewer hydrogen bonds with water.^86^ Trehalose is most often compared to sucrose – another non-reducing disaccharide. The two have a similar structure; trehalose being made up of two glucose subunits and sucrose of one glucose and one fructose subunit. They have equal molecular weights and an equal number of hydrogen bond donors and acceptors.

However, there are a number of ways in which trehalose appears to be a better cryoprotectant than sucrose: in solution, trehalose forms stronger hydrogen bonds with water than sucrose,^87^ and becomes associated with more molecules of unfrozen water, in this way each molecule of trehalose is able to disrupt the hydrogen bonding network of a greater number of water molecules, preventing them from forming ice.^88,89^ Trehalose has superior ice growth inhibiting properties compared with sucrose.^90^ Unlike sucrose, trehalose is able to displace water bound to the carbonyl groups of phospholipid membranes, displacing more water.^91^ Trehalose has a larger hydrated volume than sucrose, which is correlated with protein protection, and more sucrose is needed to provide the same level of protein protection as trehalose.^21,92^ Trehalose and sucrose cause a dynamic slowing effect on the surrounding water network, this effect is stronger in trehalose.^93^ Some of these effects may occur because sucrose has more intramolecular hydrogen bonding, resulting in fewer and/or weaker intermolecular hydrogen bonds to interact with water.^88^ Trehalose solutions have a higher glass transition temperature than sucrose solutions,^94,95^ however, a lower concentration of sucrose is needed to vitrify in aqueous solution at any given cooling rate.^96^ During freeze-drying where water content is very low and samples are stored at room temperature, trehalose would be the superior stabiliser as it would be more likely to remain above its glass transition temperature, even if it absorbed a small amount of water. Conversely, during cryopreservation, sucrose may be more useful (at least with regard to its vitrifying properties) in both vitrification, and in slow freezing where vitrification occurs due to freeze concentration. Sucrose also has greater solubility in water,^97^ making it less likely to precipitate out of solution at high concentrations and low temperatures. In some organisms that use trehalose as a cryoprotectant, antifreeze proteins may be used to mitigate this solubility problem.^98^ A summary of the comparative advantages of trehalose and sucrose can be seen in Table 1. While trehalose is – in theory – a better cryoprotectant than other sugars, the results of experimental cryopreservation studies where trehalose has been directly compared to other sugars are mixed. Discussed below are cryopreservation studies where trehalose has been tested and found to be a better, worse, or equivalent cryoprotectant compared to other sugars.

**Table 1:** Comparison of the advantages of trehalose *versus* sucrose for cryopreservation

Advantages of trehalose	Advantages of sucrose
Higher *T*_g_ at any given concentration (106 °C *vs.* 60 °C for the pure sugars)^95^	Lower *C*_v_ at any given volume (74% *vs.* 58% at 20 μl)^96^
Higher hydration number (4.598 *vs.* 4.127 at 66% w/v)^88^	Greater solubility (68.1% *vs.* 52.3% at 30 °C)^97^
Larger hydrated volume (62.5% *vs.* 87% water for a 1.5 M solution)^21^	Low concentration required to vitrify at a given cooling rate^96^
Superior ice growth inhibiter (“approximately twice as effective”)^90^	—
Displace water bound to the carbonyl groups of phospholipid membranes^91^	—
Causes more dynamic slowing of the surrounding water network^93^	—
More effective for protein stabilisation^92^	—

#### Studies where trehalose outperforms other sugars

Cryopreservation of human embryonic stem cells with 10% DMSO + 200 mM trehalose, and adding trehalose to the recovery media, resulted in a greater number of undifferentiated cells surviving compared to DMSO alone. No improvement was seen with sucrose.^99^ In human amniotic fluid stem cells, trehalose was slightly better than sucrose in the absence of solvent, although both were inferior to standard cryoprotectants.^100^ In bovine spermatogonial stem cells, 20% DMSO + 200 mM trehalose improved stem cell recovery relative to DMSO alone, DMSO + sucrose, or to DMSO + PEG (19% *vs.* 7% *vs.* 5% respectively). The cells preserved with trehalose also showed the lowest post-thaw apoptosis.^101^ Trehalose was also better than sucrose for mouse oocyte vitrification,^102^ and improved post-thaw outcome relative to sucrose in human spermatozoa.^103^ In ram spermatozoa, trehalose was superior than sucrose and slightly better than raffinose at 100 mM.^104^ The presence of trehalose or sucrose in a cryoprotectant solution reduces solvent cryoprotectant toxicity to RBCs, but this effect is higher for trehalose.^26^ In mouse neuroblastoma cells, trehalose gave higher post-thaw survival than sucrose (35% *vs.* 20%). Cells frozen in trehalose benefit from trehalose preincubation, whereas cells frozen in sucrose do not benefit from preincubation with sucrose. The most effective cryoprotectant combination was trehalose + l-proline in a 1 : 1 ratio, at a total concentration 112.5 mM. This gave a post-thaw survival of 51%.^105^ Inositol is a non-reducing monosaccharide that can confer freeze tolerance to cricket cells, but not to crickets, while trehalose can confer freeze tolerance to both cricket cells and to whole crickets.^106^ Inositol is inferior to trehalose for protection during lyophilisation.^107^

#### Studies where other sugars outperform trehalose

Multiple sugars (trehalose, sucrose, sorbitol, glucose and mannitol) were tested for the cryopreservation of *Catharanthus roseus* cells, it was found that trehalose resulted in the lowest survival rate and sorbitol gave the highest.^108^ Sucrose was marginally better than trehalose for post-thaw survival in saltwater crocodile spermatozoa preservation.^109^ Mentioned above, Chaytor *et al.*^84^ measured the IRI activity of six different sugars (galactose, glucose, melibiose, lactose, trehalose, sucrose). They found that 220 mM lactose has the best IRI activity. Viability in a 200 mM solution of these sugars was then measured in human hepatocytes, both after incubation at 37 °C, and after freeze–thaw. Galactose and lactose gave the best viability after incubation at 37 °C. Galactose gave the best post-thaw viability, while glucose gave the worst. Trehalose was not better than sucrose. While this study does show that the selection of sugar can affect post-thaw outcome, the differences in post-thaw survival were not large (with the exception of glucose, which was by far the worst cryoprotectant here). Another study assessed the effect of multiple sugars (lactose, galactose, glucose, fructose, lactulose, melibiose, trehalose, sucrose) on post-thaw boar sperm quality. It was found that disaccharides were better than monosaccharides, and those disaccharides that contain glucose were better than does which did not. Trehalose increased membrane permeability in this study, resulting in worse post-thaw outcome than other sugars.^84^

#### Studies where trehalose and other sugars were equivalent

Rat hepatocyte viability after cryopreservation was slightly higher (80% *vs.* 75%) with 200 mM trehalose in the absence of DMSO, than with 5% DMSO. The addition of trehalose or sucrose to 5% DMSO did not improve viability, and the addition of glucose decreased viability.^33^ In mouse spermatogonial stem cells, trehalose did not significantly increase recovery, but almost doubled post-thaw proliferation. Trehalose was more effective than sucrose and maltose in this respect. Lactose was as effective as trehalose. Fructose, galactose, xylose, and raffinose did not increase proliferation compared to the control. Glucose and mannose did increase proliferation, but not as much as the disaccharides.^83^ In Rodrigues *et al.*^31^ post-thaw recovery with 5% DMSO + 146 mM trehalose was 85%, compared to 79% recovery with 10% DMSO for the preservation of human cord blood hematopoietic stem cells. Although there was no difference between using trehalose and sucrose in this study. Nynca *et al.*^110^ reports that sperm cryopreservation can be improved with glucose, sucrose, or trehalose, but trehalose was not more effective than other sugars for most Salmonidae species. The addition of 250 mM trehalose or 321 mM lactose to boar sperm freezing extender had no beneficial effects.^111^ In Woelders *et al.*^112^ bull sperm, isotonic trehalose and sucrose were equally beneficial at the optimal fast cooling rate, and in Chen *et al.*^113^ trehalose and sucrose provided equal benefit to bull spermatozoa motility. For the vitrification of human spermatozoa, trehalose and sucrose were equally effective at preserving motility, and were both slightly better than 3-*O*-methyl-d-glucopyranose, raffinose and stachyose.^114^ In dog spermatozoa vitrification, trehalose was not more effective than sucrose.^115^

## Trehalose as an intracellular cryoprotectant

As discussed above in the context of cryopreservation performance, trehalose is most effective when it is in both the intracellular and extracellular space.^116–118^ In the intracellular space, it is able to inhibit intracellular ice, prevent cellular dehydration caused by both extracellular ice and extracellular cryoprotectants, and stabilise intracellular proteins. However, trehalose is a polar molecule, and does not cross the cell membrane by diffusion, nor are there any trehalose-specific transporters in most mammalian cells (*e.g.* a non-penetrating cryoprotectant). Various methods have been used to load trehalose into the intracellular space of cells which do not naturally produce it namely; electroporation, membrane permeabilising polymers, genetic engineering, microinjection, nanoparticles, liposomes, freeze-induced uptake, molecular engineering, and natural endocytosis through preincubation (Fig. 5). For an in-depth review of the topic, see Stewart and He.^119^ The ideal method for trehalose permeation would have low cytotoxicity, not be time-consuming or labour-intensive, and would allow for a high enough concertation of trehalose to be loaded that it could entirely replace penetrating cryoprotectants.

### Preincubation, fluid-phase endocytosis, and osmotic manipulation

Fluid-phase endocytosis, or “cellular drinking” is where cells internalise the surrounding fluid using vesicles. This allows for the non-specific uptake of molecules from the surrounding environment. In this way, cells can be slowly loaded with trehalose by incubating them in a trehalose solution for a number of hours. Trehalose pre-incubation was first used in carrot and tobacco cells where it allowed for trehalose to be used as the sole cryoprotectant.^120,121^ This process is slow – cells have to be incubated for up to 24 hours before freezing – and the maximum concentration of trehalose that can be loaded is limited. Preincubation with trehalose has been used to improve the cryosurvival of mesenchymal stem cells,^122^ and is more effective than simply adding trehalose to the cryopreservation media without preincubation.^123^ In Stokich *et al.*^124^ human hepatocyte cell monolayer recovery was improved by a 24-hour preincubation with 100 mM trehalose in addition to the standard 10% DMSO, resulting in 39% and 10% viability respectively. In Campbell *et al.*^125^ preincubation with 200 mM trehalose before preservation with 400 mM trehalose greatly increased metabolic activity measured 7 days after thawing in bovine endothelial cells. In Hara *et al.*^126^ 1.3 M trehalose in the absence of solvent increased proliferation compared to glycerol for human embryonic kidney cells (36% *vs.* 10%). For cattle ovarian granulosa, a 30-minute preincubation with 200–400 mM trehalose, before flash freezing with trehalose + 5% DMSO and 5% EG, greatly improved viability (70% *vs.* 15%).^127^ It is possible to force trehalose to cross the cell membrane by creating large osmotic pressure differences between the intracellular and extracellular spaces. However, it only allows for small amounts of trehalose to be loaded and it can damage cells. This has been attempted in red blood cells, allowing for up to 43 mM trehalose to be loaded.^128,129^ This has also been attempted in T-lymphocytes, resulting in 2.2 mM being loaded.^130^

### Freeze-induced uptake

When trehalose is simply added to the extracellular space immediately prior to freezing, it can be mistakenly considered to only be an extracellular cryoprotectant. However, the phase change in the cell membrane during freezing, combined with freezing-induced osmotic effects and membrane damage, allows trehalose and other small molecules to enter the cell. The amount of trehalose loaded by freeze-induced uptake increases with freezing rate, resulting in a faster optimal cooling rate for cryoprotection from freeze-induced uptake than with DMSO.^131^ Beattie *et al.*^132^ was one of the earliest studies using intracellular trehalose to preserve mammalian cells. Here, human pancreatic islets were cryopreserved with 2 M DMSO + 300 mM trehalose, which resulted in improved cell recovery compared to 2 M DMSO + 11.1 mM glucose (94% *vs.* 58%). Insulin production was also greatly improved. Intracellular trehalose was measured and was found to increase as temperature was lowered. The threshold temperature for freeze-induced uptake appears to be 15 °C, as only very small amounts of intracellular trehalose were detected before this temperature was reached. In mouse neuroblastoma cells, both preincubation and freezing with trehalose contribute to increased cell recovery. Trehalose positively combines with l-proline at equal total concentration, even when l-proline was simply added to the extracellular freezing media, suggesting that l-proline protects cells through a mechanism other than inducing freeze tolerant metabolism.^105^ In Zhang *et al.*^133^ a 500 mM extracellular trehalose concentration was optimal for mouse embryonic fibroblasts, resulting in 50% survival. 160 mM trehalose could be detected inside cells after preservation. Preincubation with 25 mM trehalose had no positive effect. Freeze-induced uptake may also occur in platelets^134^ and spermatozoa.^135^

### Electroporation

Electroporation is the application of an electric current to cells which causes the formation of pores, resulting in a temporary increase in membrane permeability. In Shirakashi *et al.*^136^ mouse myeloma cells were loaded with 100 mM trehalose from an extracellular trehalose concertation of 290 mM. Higher intracellular concentrations were achievable but required damaging voltages to be used. In Dovgan *et al.*^137^ human umbilical stem cells were electroporated and incubated with 200 mM trehalose for 25 minutes, which resulted in improved cell recovery relative to 10% DMSO (86% *vs.* 60%). For human adipose-derived stem cells, electroporation with trehalose increased recovery compared to trehalose incubation, as measured by trypan blue excision but not when measured by MTT assay.^138^

### Polymers

A polymer known as ‘PP-50’ (poly(l-lysine iso-phthalamide) grafted with l-phenylalanine) has been used to increase membrane permeability in a reversible pH-responsive manner.^139^ It can be used to permeabilise human red blood cells to trehalose resulting in 251 mM trehalose uptake from 700 mM solution after a 9 h incubation period. In ovine red blood cells, PP-50 allowed 123 mM trehalose loading from a 360 mM extracellular solution, with a 9-hour incubation. This resulted in 83% survival between the end of incubation and thawing. However, PP-50 causes some haemolysis, and this was not accounted for in the post-thaw haemolysis lysis assay, as RBCs were washed after incubation, which removed free haemoglobin. Elsewhere in the paper, it was found that 22% haemolysis occurs during loading, resulting in a probable final survival of around 61%. So, while this shows that the level of trehalose loading archivable with PP-50 greatly improves survival, it does not present PP-50 as a good way of achieving it. Nonetheless, PP-50 can be used to increase cryosurvival with trehalose uptake.^116^ The PP-50 trehalose combination was also used to improve the cryosurvival of osteosarcoma cells, although does not result in better cryosurvival than that with DMSO.^140,141^

### Carrier peptides

A cargo peptide was developed by Wei *et al.* that non-covalently binds to trehalose and carries it across the cell membrane. The peptide was non-toxic, but only very small amounts of trehalose could be loaded into the cell.^142^

### Gene expression for trehalose synthesis

Trehalose can be synthesised from glucose using trehalose 6-phosphate synthase and trehalose 6-phosphate phosphatase. Cells can be transfected with genes that encode these enzymes, but the intracellular trehalose concentration that can be achieved is low.^143,144^

### Transporter proteins and channels

Most cell types have an endogenous receptor called P2Z (or P2X7) which forms pores when ATP is present extracellularly. In human hematopoietic stem cells, it allowed for 200 mM trehalose to be loaded from 200 mM extracellular trehalose after a 60-minute incubation period, resulting in 91% post-thaw viability as measured by proliferation. This is compared to 11% viability after freezing with 200 mM extracellular trehalose alone.^117^ Other pores and channels can be added artificially. For example, cells can be genetically engineered to express the trehalose transporter TRET1. In Chinese hamster ovary cells, this allowed 23 mM trehalose uptake after incubation in 400 mM trehalose for 4 hours. This did not affect the cells' proliferation.^145^ This technique was later used to protect Chinese hamster ovary cells during cryopreservation.^146^ H5 is a pore-forming agent which has been engineered from α-haemolysin. H5 forms pores in the cell membrane which can be opened and closed by altering zinc ion concentration. This allowed for 80% cryosurvival in murine fibroblasts, 70% cryosurvival for human keratinocytes,^147^ and 92% post-thaw viability for human hemopoietic stem cells.^148^ This approach allows for high concentrations of trehalose to be loaded quickly, but the potential of H5 to cause an immune response may limit its use in cells that are to be used clinically.

### Microinjection

Microinjection allows for small volumes of liquid to be directly introduced into the intracellular space. It allows very high concentrations of trehalose to be loaded but it poses challenges when used on cells which are smaller than oocytes. In Eroglu *et al.*^149^ human oocytes were microinjected with 150 mM trehalose and frozen in 500 mM trehalose, resulting in 66% survival. In a later study, mouse oocytes were injected with 500 mM trehalose and frozen in 500 mM trehalose solution, giving 80% survival. Using 500 mM extracellular trehalose only gave just 15% survival. Reducing intracellular trehalose concentration to 80 mM and adding 500 mM DMSO (4% w/v) resulted in 97% survival. These oocytes were able to develop into embryos at the highest rate. One explanation for the increased cryosurvival in the presence of DMSO, is that DMSO crosses intracellular membranes and enter the organelles.^150^

### Nanoparticles

Nanoparticles can encapsulate trehalose and deliver it into the cell. Thermally responsive nanoparticles can be used to load up to 300 mM trehalose into murine fibroblasts, from a 40 minute incubation followed by cold shock.^151^ In human adipose-derived stem cells, 24-hour incubation with pH-responsive nanoparticles and 200 mM extracellular trehalose allowed for a post-thaw viability equivalent to that given by 10% DMSO.^152^ Nanoparticles that increase membrane permeability have been used to load 51 mM trehalose into red blood cells from 350 mM extracellular trehalose, after a 7 hour incubation period, resulting in 91% post-thaw survival.^153^

### Liposomes

Holovati and Acker^154^ developed liposomes that can be used to deliver trehalose into RBCs. These liposomes were used to carry a small volume of 300 mM trehalose and were incubated with red blood cells for 4 hours (for an estimated total of 15 mM intracellular trehalose), then frozen with 300 mM trehalose. This resulted in 67% post-thaw recovery, compared to 27% recovery when incubated in extracellular trehalose alone. The concentration of intracellular trehalose alone provided minimal cryoprotection, but surprisingly the liposomes themselves provided a very large degree of cryoprotection; cells incubated with liposomes containing no trehalose and frozen in 300 mM extracellular trehalose gave 60% recovery.^155^ The ability of liposomes to improve cryopreservation could cause misleading results when using them to deliver cryoprotectants. For example, Motta *et al.*^156^ used liposomes to assess the effect of intracellular and extracellular trehalose on umbilical cord blood stem cell preservation but did not control for the effects of the liposomes themselves. However, this cryoprotective ability is useful in its own right, and could be used as part of a multi-component cryoprotectant solution to deliver low concentrations of non-penetrating cryoprotectants such as antifreeze proteins. In Stoll *et al.*^157^ liposomes allowed for up to 80 mM trehalose to be loaded into human RBCs, dependent on liposome concentration. This slightly increased post-thaw recovery, but post-thaw recovery did not increase with increasing liposome concentration above 1 mM, which was the lowest liposome concentration used.

### Ultrasound

In Zhang *et al.*^158^ ultrasound was used to induce non-specific permeability in human platelets. This technique allowed only 30 mM of trehalose to enter the cell after 30 minutes of treatment, which is insufficient for cryoprotection. This method also altered platelet morphology.

### Modified trehalose

Trehalose itself can be chemically modified so that it can penetrate the cell membrane (Fig. 6). In Abazari *et al.*^159^ trehalose was acetylated at six of its hydroxyl groups, greatly increasing its hydrophobicity. Rat hepatocytes were incubated with 30 mM acetylated trehalose, resulting in an intracellular acetylated trehalose concentration of 80 mM within 1 hour, and of up to 300 mM after 8 hours. Trehalose concentration lagged behind this with a concentration of around 100 mM after 12 hours.

### Trehalose removal

While trehalose has almost no direct toxicity at concentrations required for cryopreservation, trapped intracellular trehalose can cause osmotic damage when trehalose is removed from the extracellular solution.^131^ However, after thawing, trehalose will be eliminated from cells due to cell division, and possibly by exocytosis and/or conversion to glucose.^160–162^ Therefore, to maximise cell survival, the osmolarity of the recovery media can be progressively lowered to maintain osmotic balance, although this strategy is labour-intensive. Alternatively, using a lower intracellular concentration of trehalose in conjunction with a penetrating cryoprotectant can mitigate osmotic damage.^150^

## Conclusions

This review has summarised the broad scope of trehalose in cryopreservation, highlighting specific areas where there were successes and also where the benefits were limited. We have also gathered the literature on solutions to overcome the limitations of trehalose's low membrane permeability, to show where chemistry can be deployed to delivery this inside cells: adapting trehalose to become a penetrating cryoprotectant. Whilst trehalose clearly shows benefits when added to the extracellular media, the delivery question challenge remains open to innovative solutions which may enable the reduction or removal of the need for conventional penetrating cryoprotectants (such as DMSO) whilst ensuring that post-thaw cells are viable and functional. With the rapid growth of cell-based therapies and biotechnology process, the need for tools to bank, transport and deliver cryopreserved cells (and other biological components) has never been higher. Any emerging tools will all need critical evaluation of the price, biocompatibility, and accessibility, compared to *e.g.* DMSO which is low cost, widely available and provides consistent results.

## Data availability

This is a review article and no new data was generated.

## Conflicts of interest

There is no conflict of interest to declare.
